# A novel framework for discharge uncertainty quantification applied to 500 UK gauging stations

**DOI:** 10.1002/2014WR016532

**Published:** 2015-07-19

**Authors:** G. Coxon, J. Freer, I. K. Westerberg, T. Wagener, R. Woods, P. J. Smith

**Affiliations:** ^1^ School of Geographical SciencesUniversity of BristolBristolUK; ^2^ Department of Civil EngineeringUniversity of BristolBristolUK; ^3^ IVL Swedish Environmental Research InstituteStockholmSweden; ^4^ Lancaster Environment Centre, Lancaster UniversityLancasterUK

**Keywords:** discharge data, LOWESS, rating curve, generalized framework, observational uncertainty, United Kingdom

## Abstract

Benchmarking the quality of river discharge data and understanding its information content for hydrological analyses is an important task for hydrologic science. There is a wide variety of techniques to assess discharge uncertainty. However, few studies have developed generalized approaches to quantify discharge uncertainty. This study presents a generalized framework for estimating discharge uncertainty at many gauging stations with different errors in the stage‐discharge relationship. The methodology utilizes a nonparametric LOWESS regression within a novel framework that accounts for uncertainty in the stage‐discharge measurements, scatter in the stage‐discharge data and multisection rating curves. The framework was applied to 500 gauging stations in England and Wales and we evaluated the magnitude of discharge uncertainty at low, mean and high flow points on the rating curve. The framework was shown to be robust, versatile and able to capture place‐specific uncertainties for a number of different examples. Our study revealed a wide range of discharge uncertainties (10–397% discharge uncertainty interval widths), but the majority of the gauging stations (over 80%) had mean and high flow uncertainty intervals of less than 40%. We identified some regional differences in the stage‐discharge relationships, however the results show that local conditions dominated in determining the magnitude of discharge uncertainty at a gauging station. This highlights the importance of estimating discharge uncertainty for each gauging station prior to using those data in hydrological analyses.

## Key Points

- A generalized framework for discharge uncertainty estimation is presented
- Allows estimation of place‐specific discharge uncertainties for many catchments
- Local conditions dominate in determining discharge uncertainty magnitudes

## 1 Introduction

Gauging stations worldwide provide river discharge data for a diverse range of organizations including water authorities, government departments, academia and insurance companies. These data not only underpin our knowledge of the hydrologic system but they are also utilized for flood and drought analysis, hydrological modeling, and water resources monitoring, from which important management decisions are made. Therefore, it is essential that the quality of discharge data is assessed and benchmarked [*McMillan et al*., 2012] to ensure robust decision making and obtaining the right answers for the right reasons [*Kirchner*, 2006]. Many studies have pointed to the importance of accounting for uncertainties in discharge data for hydrological modeling analyses [*Westerberg et al*., 2011b; *Coxon et al*., 2014; *Kuczera et al*., 2010; *Liu et al*., 2009; *Krueger et al*., 2010; *McMillan et al*., 2010], flood modeling [*Pappenberger et al*., 2006; *Domeneghetti et al*., 2012], flood frequency estimation [*Kuczera*, 1996], nonideal flow conditions [*Birgand et al*., 2013], and water‐level predictions [*Sikorska et al*., 2013]. Quantifying discharge uncertainty magnitude and its characteristics is an important step in these studies as discharge uncertainties can be significant and change over time because of changes in channel shape due to weed growth, sedimentation or erosion [*Tomkins*, 2014; *Westerberg et al*., 2011a; *McMillan et al*., 2010] or from extrapolation of the rating curve [*Kuczera*, 1996].

Techniques for quantifying the uncertainty in discharge data are becoming an increasingly important area of scientific research within hydrology [*McMillan et al*., 2012] and are currently a major challenge for hydrologic science. Limited access to gauging data, rating curves and gauging station metadata—including information about how the stage‐discharge measurements are collected and site conditions—hampers their more widespread use. This also means that it is often difficult for hydrologists to reconcile the different sources of uncertainty into measures of discharge uncertainty through time and across the flow range for a gauging station. Moreover, errors in the stage‐discharge relationship arise from a mix of both random (aleatory) and nonrandom (epistemic) errors, which can often be difficult to characterize and quantify [*Beven et al*., 2011]. As a result, there are many different methods to assess the uncertainty in the stage‐discharge relationship with recent studies focusing efforts on quantifying discharge uncertainty for different gauging stations and types of stage‐discharge relationships. Traditional statistical approaches [e.g., *Venetis*, 1970; *Clarke*, 1999; *Clarke et al*., 2000; *Petersen‐Øverleir and Reitan*, 2005] base their discharge uncertainty bounds on the residual variance from a regression function or the variance of the parameter estimates for the rating curves, but do not explicitly incorporate measurement uncertainty in the gauging data. Bayesian approaches [e.g., *Moyeed and Clarke*, 2005; *Petersen‐Øverleir and Reitan*, 2009; *Reitan and Petersen‐Øverleir*, 2009; *Le Coz et al*, 2014; *Juston et al*., 2014] offer the advantage of incorporating hydraulic knowledge of the gauging station to set informative priors on the rating‐curve parameters and deriving a likelihood function that accounts for uncertainty in the individual stage‐discharge measurements [e.g., *Le Coz et al*., 2014]. Alternative approaches [e.g., *Krueger et al*., 2010; *Guerrero et al*., 2012; *Jalbert et al*., 2011; *Westerberg et al*., 2011a; *McMillan et al*., 2010] have tended to concentrate on non‐stationary rating curves where the typical assumptions made in statistical approaches may result in biased uncertainty estimates. These studies have utilized different approaches to estimate discharge uncertainty from fitting multiple rating curves to subsets of the data [*McMillan et al*., 2010], using fuzzy regression [*Westerberg et al*., 2011a; *Shrestha et al*., 2007] and variographic analysis [*Jalbert et al*., 2011]. *McMillan and Westerberg* [2015] developed a likelihood function that accounts for both random and epistemic errors and enables sampling of multiple feasible rating curves. There are also opportunities to exploit flood routing and inundation modeling to estimate discharge uncertainties, particularly for out of bank flows, where there are typically a lack of stage‐discharge measurements [*Di Baldassarre and Claps*, 2010; *Di Baldassarre and Montanari*, 2009; *Franchini et al*., 1999]. However, all of the aforementioned studies have been applied to a limited number of case studies or to a single type of stage‐discharge relationship. One notable exception is *Petersen‐Øverleir et al*. [2009] who utilize a Bayesian approach to calculate 95% credibility intervals for 581 gauging stations in Norway. They find that many of the stations in Norway fail to meet WMO and ISO 1992 criteria of discharge uncertainty between 5 and 8%. However, the assumptions behind their method are not suitable in all cases, e.g., for stations with significant epistemic error in the model representation of the stage‐discharge relationship. Hence, there remains a lot of scope for expanding our knowledge and understanding of how discharge uncertainty varies between gauging stations and across discharge ranges.

This paper aimed to develop a novel generalized framework to quantify, characterize and benchmark discharge uncertainty for hundreds of gauging stations. The framework was demonstrated on rating curves and stage‐discharge measurements from 500 gauging stations across England and Wales, which represent a range of errors and different types of stage‐discharge relationships. The framework is primarily based on a nonparametric regression and includes methods for determining gauging measurement uncertainty, for using information from the temporal changes in the rating curves to pool groups of stage‐discharge measurements, and for modeling multisection rating curves. Results from the study were used to: (1) examine whether such a general methodology can estimate place‐specific uncertainties, and (2) benchmark discharge uncertainties (i.e., provide a benchmark measure of discharge uncertainty across England and Wales from which other gauging stations can be measured or judged against) at a national scale.

## 2 Methods

### 2.1 Study Area and Data Availability

**Figure 1:**
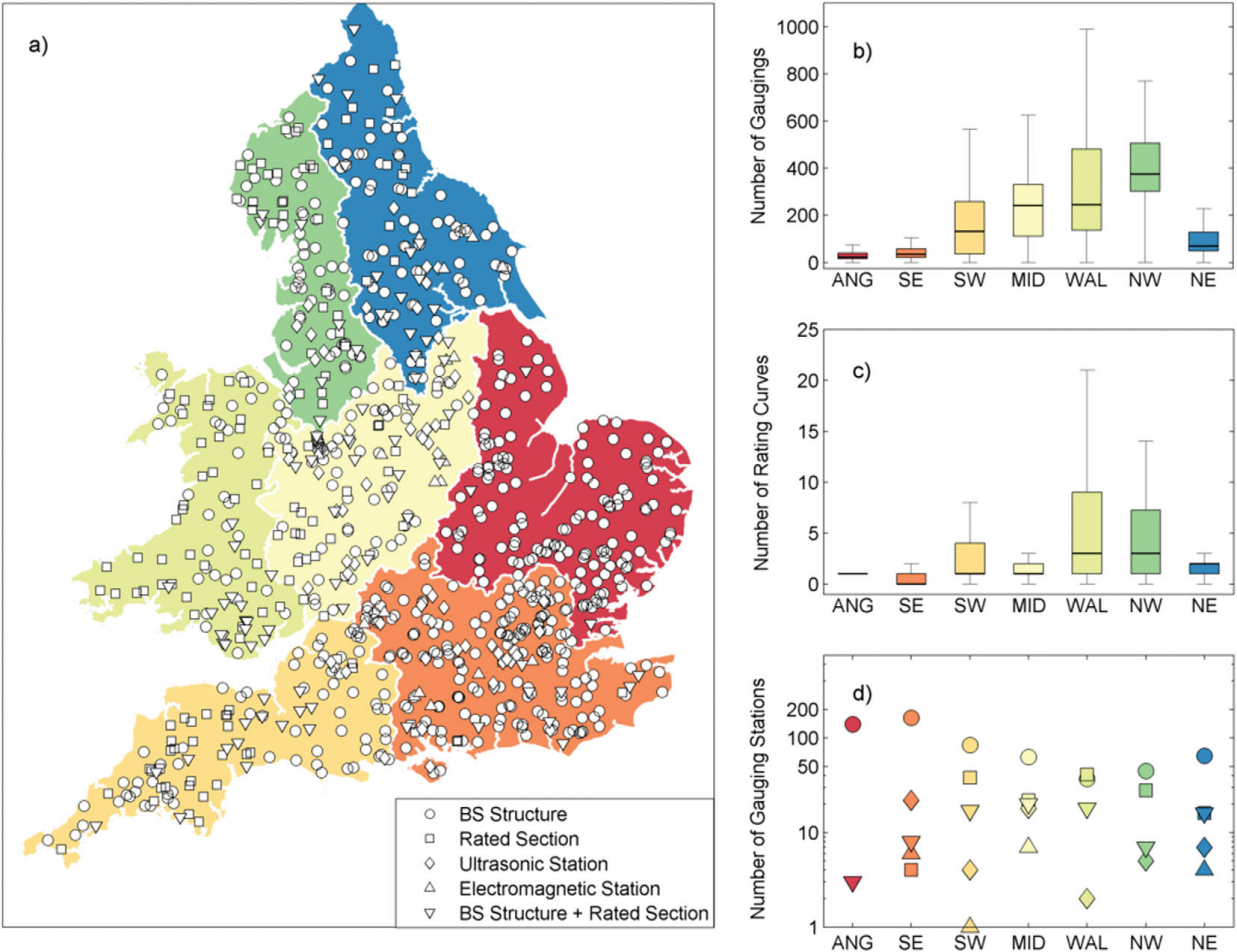
(a) Gauging station types in England and Wales for 2008, (b) number of stage‐discharge measurements per gauging station, (c) number of historical rating curves per gauging station, and (d) number of different gauging station types in each region. *BS structure—A structure (any form of weir or flume) operated to British Standard. **ANG—Anglian, SE—South East, SW—South West, MID—Midlands, WAL—Wales, NW—North West, NE—North East.

We chose 500 gauging stations to evaluate the framework for discharge uncertainty estimation from a large data set of 910 gauging stations located throughout England and Wales (Figure 1a). For each gauging station, all rating curve and stage‐discharge measurement data (if available) were obtained from the Environment Agency (EA). The total number of stage‐discharge measurements for a single gauging station varied from 0 (where only a theoretical rating curve was available) to 1797, with the earliest stage‐discharge measurement on record obtained in 1955 and the most recent measurements obtained in 2013. The number of different historical rating curves varied from 0 to 89 for each gauging station. This dense gauging station network represents a diverse range of conditions in terms of climate, topography, land use and geology within England and Wales. Regional hydrometry teams within the EA are responsible for the day‐to‐day operation and maintenance of these stations, including taking stage‐discharge measurements and making decisions on when to change the rating curves. A wide range of gauging stations are operated across the regions including purpose‐built gauging structures, rated open channel sections, ultrasonic and electromagnetic flow measurement methods or a combination of these (Figure 1). Figure 1 highlights the regional differences in the gauging network. The South and East of England is characterized predominantly by weirs and flumes, small numbers of stage‐discharge measurements and usually only one rating curve. Conversely, Wales, the South West and North‐West of England is characterized by a mix of gauging stations with many more rated sections, high numbers of historical rating‐curve changes and a corresponding large number of stage‐discharge measurements. Thus, the data set provides a challenge as any discharge uncertainty analysis will need to account for a wide range of different and complex stage‐discharge relationships.

Metadata was taken from the UK Hydrometric Register [*Marsh and Hannaford*, 2008] and the Centre for Ecology and Hydrology National River Flows Archive (NRFA) website (http://www.ceh.ac.uk/data/nrfa). The metadata included information on the type and history of the gauging station, information about problems associated with weed growth or siltation, and bankfull stage. Data on the field methods used to collect the stage‐discharge measurements were not available for every gauging station and neither was detailed information on the hydraulic characteristics of each site (e.g., channel geometry, channel roughness and change in section controls).

### 2.2 Discharge Uncertainty Sources in England and Wales

**Figure 2:**
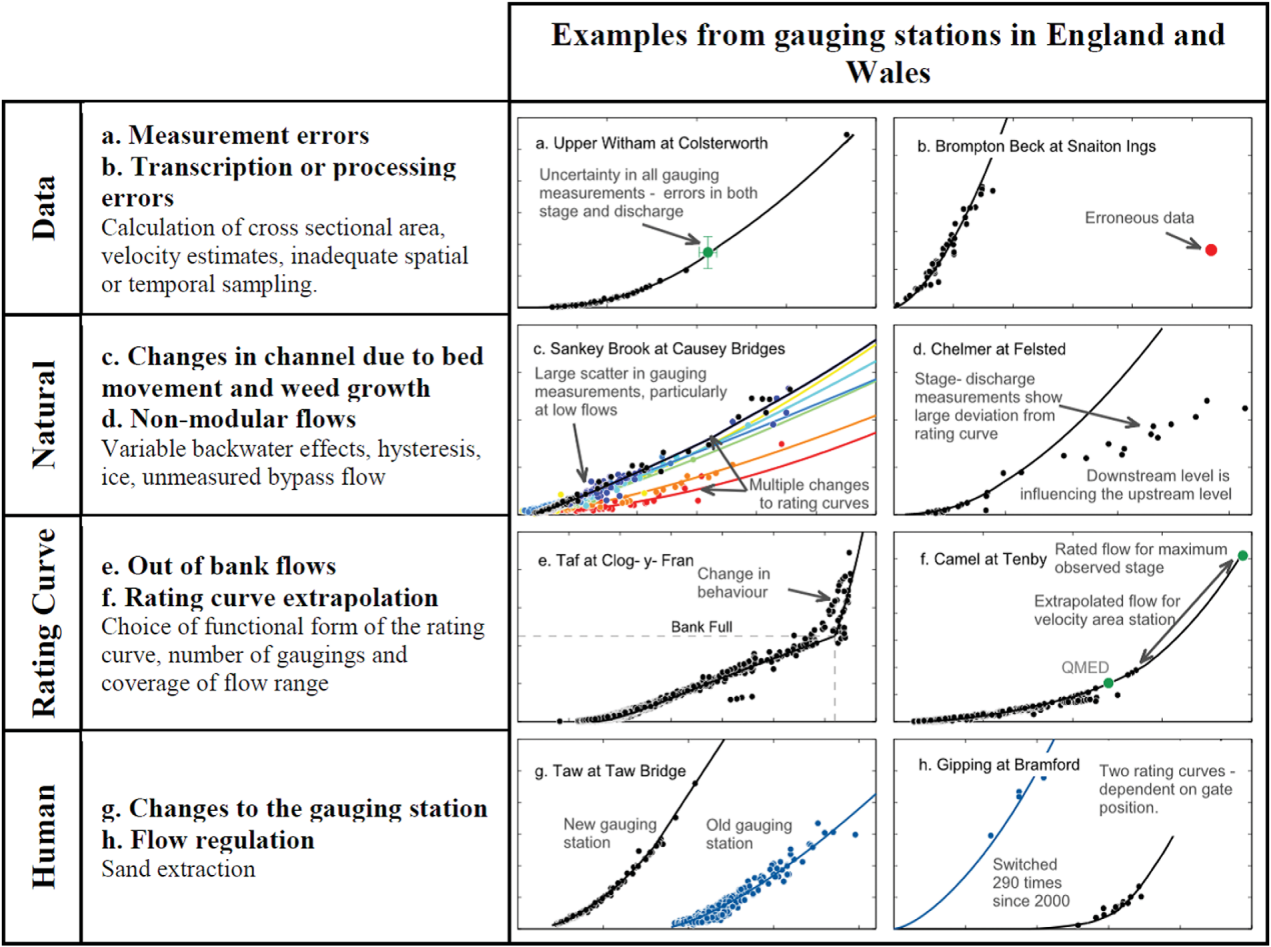
Uncertainties in rating‐curve modeling of the stage‐discharge relationship with examples from gauging stations in England and Wales. *QMED—Median annual maximum flood.

The majority of river discharge time series in England and Wales are derived from a multisection rating curve equation describing the relationship between stage and discharge. In almost all cases, each section of the rating curve is an empirical equation which takes the form of:

(1) $$Q=a{\left(h-h_{0}\right)}^{b}$$

where *Q* represents the discharge, *a* is a coefficient, *b* is an exponent, *h* is the stage and *h_0_* is the stage of zero flow. The rating curve equation is either based on theoretical weir equations where the parameters *a* and *b* are related to the shape of the weir or these parameters are calibrated from a set of stage‐discharge measurements. The use of this rating curve implies the assumption of a unique stage‐discharge relationship at the river cross section where the geometry of the section is assumed to be stable over time and where the relationship can be expressed by a single or by multiple power‐law equation(s). These assumptions are not always fulfilled and the discharge estimates can be subject to uncertainty from four main sources of error; (1) gauging data, (2) natural conditions, (3) rating‐curve approximation, and (4) human alterations. Figure 2 illustrates these four different sources of uncertainty inherent in rating‐curve estimation with examples from gauging stations in England and Wales of how these uncertainties manifest themselves in the stage‐discharge data.

Errors in the stage‐discharge gauging data are affected by both random and non‐random errors including errors in the point velocity measurements, insufficient sampling of velocity in the cross‐section, data processing errors, and level observation errors (for a comprehensive overview, see *McMillan et al*. [2012]). Nonrandom errors also arise from natural processes including erosion, sedimentation, hysteresis and weed growth, which affect the channel characteristics and hence the discharge estimates by changing the form of the stage‐discharge relationship. As a result, there may be significant scatter in the gauging data and a non‐stable relationship between stage and discharge which could be seasonal or trending over time [*Jalbert et al*., 2011; *Guerrero et al*., 2012]. The fit of the rating‐curve equation (Eqn. (1)) can also be a significant source of epistemic uncertainty (i.e., uncertainty related to lack of knowledge about the true form of the stage‐discharge relationship). Many rating curves for weirs are based on standard weir equations for which the coefficients often need calibration to gauged data. For a rated section the uncertainty in the rating curve depends on the best fit to the gauged points and thus the number and coverage of points over the flow range. This is particularly important at high flows where there are often few stage‐discharge measurements and extrapolation of the curve beyond the highest measured point is needed, thus making assumptions about the continuing form of the rating curve. In addition, there can be multiple section breaks where the rating curve can change abruptly because of different channel or hydraulic controls. Finally, human regulation and intervention can also affect the discharge uncertainty either through changing the gauging station (in England and Wales this usually means changing the gauging station from a rated section to a weir), or through the use of multiple weirs at a station (see Figure 2h) where the rating‐curve changes depending on which weir is in operation. In the calculation of discharge time series the uncertainties in stage records should also be considered. These will depend on the type of gauging station, with artificial structures designed to provide more stable and sensitive controls than natural sections.

### 2.3 A Generalized Framework for Discharge Uncertainty Estimation

**Figure 3:**
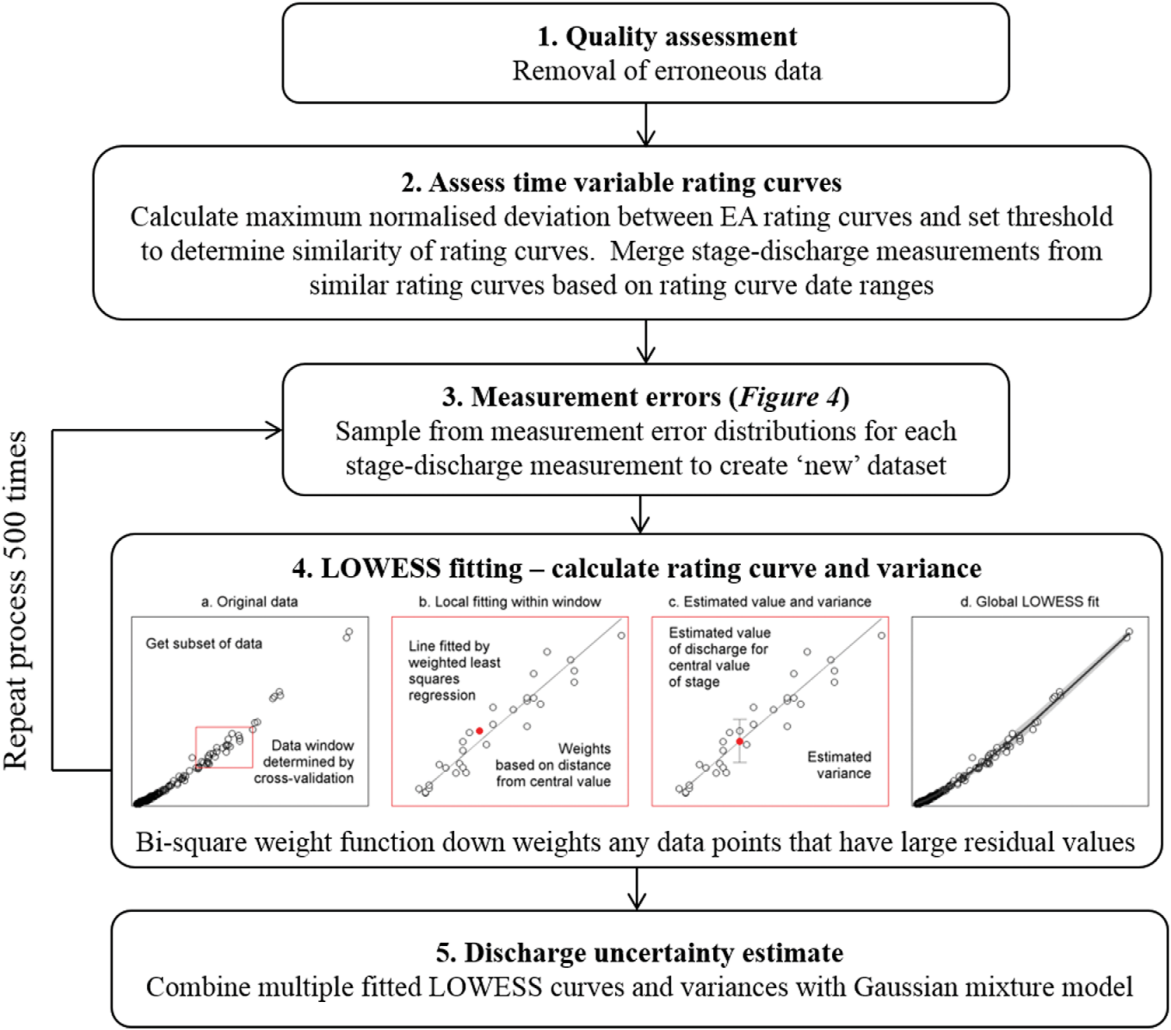
Flowchart detailing the generalized framework for discharge uncertainty estimation.

Given the wide range of place‐specific uncertainties and the limited data available on site‐specific conditions in the data set, a generalized and flexible framework was needed for the estimation of discharge uncertainty. We focused on the most prevalent uncertainties found in the data set of gauging stations from England and Wales; including errors in the stage‐discharge measurements, changes in the rating curves over time, outliers in the data and the definition of multisection rating curves. An overview of the methodology is given in Figure 3 with further description of each of the steps below.

#### 2.3.1 Gauging Data Quality Assessment

The first step was a quality assessment of the stage‐discharge data and the removal of any assumed erroneous data (Figure 3, step 1). This included stage‐discharge measurements with exceptionally high stage values (>50 m) as these had been mistakenly linked to the wrong vertical datum and no correction was possible with the metadata used in this study. Only complete stage‐discharge measurements marked as ‘good’ in the EA database were used in the analysis, however it is worth noting that the application of this marker varies by region and also was not utilized for older stage‐discharge measurements.

Discharge uncertainty estimates could be calculated for all of the England and Wales gauging stations that had stage‐discharge measurements. However, to keep the scale of the task feasible and to ensure robust discharge uncertainty estimates, we selected 500 gauging stations from the 910 gauging stations in the database. These gauging stations were selected to have more than 20 stage‐discharge measurements and an applicable rating curve (this discounted ∼32% (295) gauging stations from the total data set). A threshold of twenty stage‐discharge measurements was chosen as the nonparametric framework relies on data and fewer than 20 stage‐discharge measurements often resulted in unrealistic uncertainty bounds and/or poor LOWESS fits (see supporting information). We selected 500 gauging stations from the remaining 615 gauging stations for which there was sufficient information available about gauging data quality and the stage‐discharge relationship to ensure that the discharge uncertainty was reliably estimated. We also ensured that the selected gauging stations covered a wide range of gauging station types, locations and errors in the stage‐discharge relationship, thus providing a representative picture of the gauging network in England and Wales.

#### 2.3.2 Assessing Time Variability in the Gauging Data

An important consideration for this study was how to account for time variability in the gauging data and thus temporal changes in the discharge uncertainty. The EA accounts for changes in the stage‐discharge relationship (through weed growth, erosion, sedimentation, a new gauging station etc.) by changing the rating curve, which means that the discharge uncertainty calculated for each individual EA rating curve is different. To reflect these changes, we separated the stage‐discharge measurements into groups based on the date range for which each official EA rating curve was valid. However, this resulted in a number of groups with very low numbers of stage‐discharge measurements or a lack of stage‐discharge measurements for a particular flow range (often high flows). To allow for discharge uncertainty to be calculated for as many rating curves and flow ranges as possible, an automated method was required to pool the stage‐discharge measurements based on the similarity of the rating curves. This was seen as the most pragmatic approach given the other uncertainties. Here, we took the approach of quantifying the maximum standardized deviation between the EA rating curves for each gauge. We generated 1000 stage values between the minimum and maximum stage from all the EA rating curves, then calculated the discharge from each of the EA rating curves from this stage range and normalized the discharge values by the maximum flow. We then compared the normalized discharge values for each rating curve and if the maximum deviation between these normalized discharge values was less than 0.01 across the whole flow range we treated them as being effectively the same. In this case we then pooled the populations of individual stage‐discharge measurements together into groups of stage‐discharge measurements which would either be associated with one or multiple rating curves. This was repeated sequentially in time for all historical rating curves for each gauge.

#### 2.3.3 Quantifying Gauging Data Uncertainties

**Figure 4:**
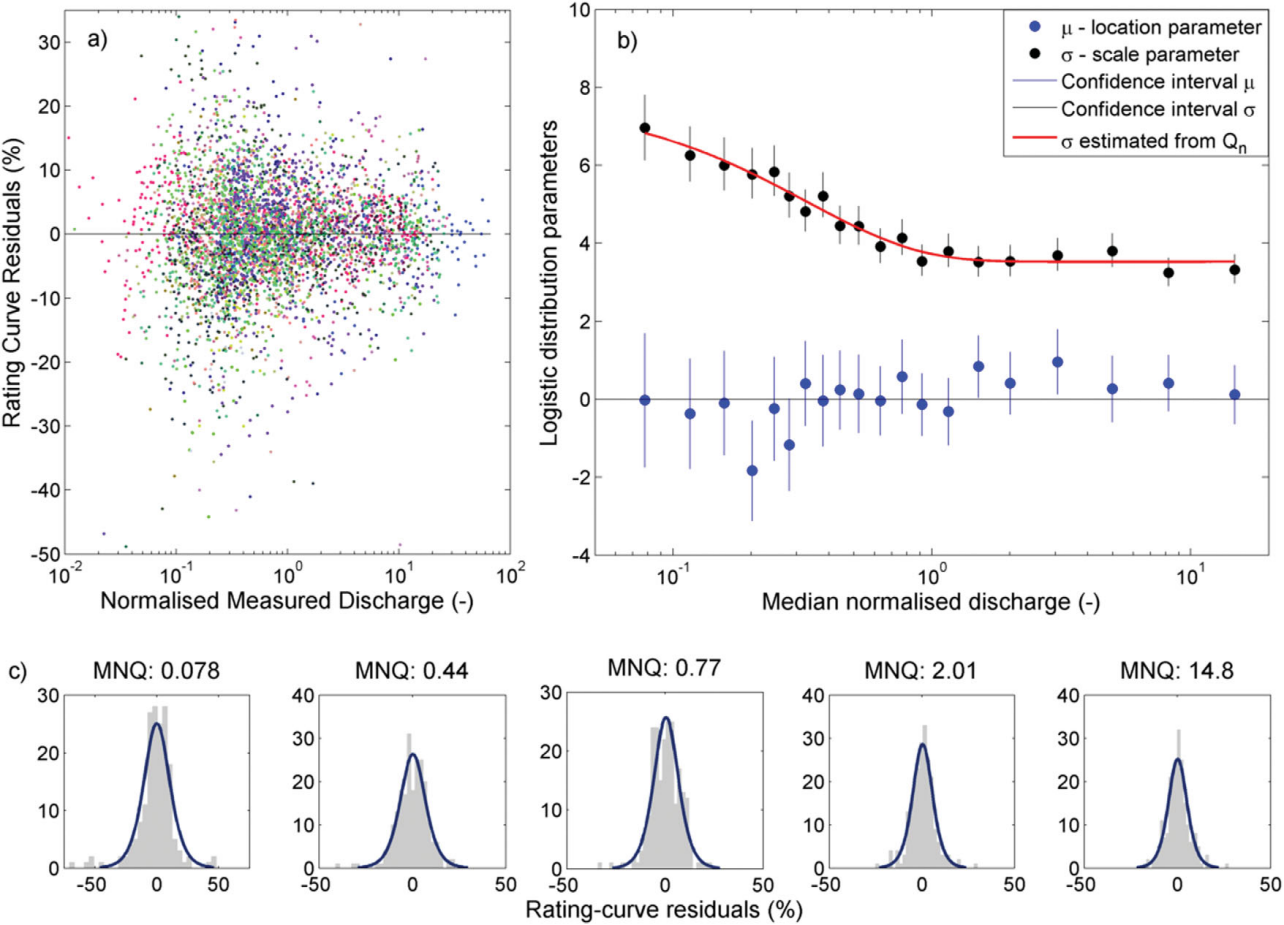
Estimation of discharge measurement uncertainty: (a) rating‐curve residuals for the 26 stable gauging stations (one colour per station), (b) logistic distribution parameters with 95% confidence intervals fitted to the residuals in 20 bins and plotted against median normalized discharge in each bin. The location parameter was approximated with zero, and the scale parameter, σ, was fitted with the function σ = 4.18 * $e^{\left(-3.051^{*}Q_{n}\right)}$ + 3.531, where *Q_n_* is normalized discharge, and (c) examples of the rating curve residuals and fitted logistic distributions for five bins in different flow ranges. *MNQ = median normalized discharge (normalized by mean flow).

To define the error associated with each discharge gauging measurement we utilized the residuals from 26 gauging stations with stable stage‐discharge relationships to estimate the measurement error as a function of normalized discharge (normalized by mean flow). These gauging stations were selected according to the following criteria; 1) no changes to the rating curve over time had occurred, 2) no outliers, and 3) marked as a good quality gauging station in the metadata from the EA. The stations covered all the hydrometric regions of the data set and measurements taken from 1962 to 2012. The majority of the stations (16, or 60%) had flow measurement structures with theoretically developed ratings for which the gauging measurements are used to check the theoretical rating. The other stations consisted of eight rated sections and two ultrasonic stations, and there was no significant difference to the estimated uncertainties if only the structures were used. The relative rating‐curve residuals (i.e., the deviation as a proportion of the measured discharge) from the 26 gauging stations were plotted against normalized measured discharge (Figure 4a). The residuals were binned into 20 intervals of normalized flow and we found that logistic distributions fitted the residuals well within each bin (Figure 4c). The parameters of the logistic distributions were plotted against the median normalized discharge in each bin (Figure 4b), and we found that the location parameter could be approximated by 0 and the scale parameter could be estimated as an exponential function of normalized discharge. The measurement errors were then sampled within the 95% limits of the logistic error distributions, which varied from ±25% for low flows to ±13% for the highest normalized flows. Note that the logistic distribution has higher kurtosis (heavier tails) than a normal distribution, which means that the corresponding 80% limits were ±15% and ±8%. The magnitude of the measurement error was inversely proportional to the normalized discharge (Figures 4a and 4b, note the *x* axis log scale), which is expected given higher relative uncertainties in low‐flow discharge measurement as a result of greater inaccuracies of hydro‐acoustic and current meter measurements in shallow and low‐velocity flow situations [*Petersen‐Øverleir et al*., 2009]. The estimated magnitudes were in line with those given by *McMillan et al*. [2012] and *Pelletier* [1988], and are expected to provide a more robust estimate of measurement error for our data set than the common practise of using a single literature estimate transferred from other studies.

Stage is measured directly and measurement errors are generally reported to be less uncertain than those for discharge measurement [*McMillan et al*., 2012]. It is likely that stage errors vary temporally because of instrumentation changes, but since we did not have any direct information on the expected stage errors we used a uniformly sampled error of ±5mm based on previous studies [*McMillan et al*., 2012].

#### 2.3.4 Fitting the Rating Curve With Nonparametric Regression

Given the wide range of stage‐discharge relationships, the large number of gauging stations, and the metadata we had for each gauging station, we needed a methodology that was (1) computationally fast, (2) ran automatically for each gauging station with minimal user input, and (3) relied only on the information from the stage‐discharge measurements. As a result of these requirements, we decided not to fit power‐law equations and instead use a nonparametric local weighted regression (LOWESS) approach [*Cleveland*, 1979] that inferred the shape of the rating curve directly from the stage‐discharge measurement data (Figure 3, step 4). The LOWESS approach was chosen because of its ability to readily compute uncertainty bounds around the predictions, to adapt to differing spacing in the stage‐discharge measurements through the use of k nearest points and the easily interpretable results.

The LOWESS procedure takes in turn each stage‐discharge measurement as the central point x in a set of 2k+1 data points. The remaining 2k data points consist of the k preceding and k following points when the data set is sorted by stage. The estimate of the discharge for the data point and its variance is generated by fitting a weighted linear regression to the selected data. This process was then repeated for all of the data points to provide a single LOWESS fit. Weights w_i_ are dependent upon the differences in stage and given by the tri‐cube weight function:

(2) $$w_{i}=\;{\left(1-\;{\left|\frac{\left(x-x_{i}\right)}{max(x-x_{i})}\right|}^{3}\right)}^{3}$$

where x is the central stage‐discharge measurement point and x_i_ are the other stage‐discharge measurements in the set of 2k+1 data points defined by the span. The weight function gives the most weight to data closest to the central stage‐discharge measurement and the least weight to the data furthest away. To account for outliers in the stage‐discharge relationship, the original data were used for a first LOWESS fit and the residuals r were then calculated from this initial fit. A bi‐square weight function was then used to weight each stage‐discharge measurement depending on how far the point is from the fitted line.

(3) $$w_{i}=\;{\left(1-\;{\left(r_{i}-\;6MAD\right)}^{2}\right)}^{2}$$

(4) MAD = median (|r|) 

where r_i_ is the residual of the i_th_ residual data point and MAD is the median absolute deviation of the residuals. This function down‐weighted any data points that had large residual values.

The span defined by k is an important consideration as large spans will result in excessively smooth curves and small spans will result in overfitting the data. Fixing the span to a set number or percentage of stage‐discharge measurements for all the gauging stations gave inconsistent results, thus the span for each set of stage‐discharge measurements was estimated. A range of spans was evaluated (0.025–25% of the number of stage‐discharge measurement points in 0.005% increments) and the span which maximized the log‐likelihood of the data for predictions generated from a leave‐one‐out cross‐validation was chosen.

#### 2.3.5 Calculating the Discharge Uncertainty Bounds

We repeated the LOWESS fit 500 times, and for each fit all the stage‐discharge measurements were resampled using the measurement error distributions described in section 2.3.3. We found that 500 times was computationally efficient and tests showed that increasing the number of samples did not change the final estimated 95% conditional uncertainty bounds. At each stage value we had 500 predictions summarized by their mean and variance, with each prediction having an equal weight. To combine the different distributions into the final uncertainty distribution for each stage value, the data distribution was expressed as a mixture of multiple Gaussian distributions whose mean and variance were derived from the LOWESS predictions for that point. We then computed the 2.5th and 97.5th percentiles of this Gaussian mixture model to define the final 95% conditional uncertainty bounds.

### 2.4 Discharge Uncertainty Results

We quantified discharge uncertainty at low, mean and high flow to compare results for each gauging station, and to compare to *Petersen‐Øverleir et al*. [2009], who estimated discharge uncertainty for 581 Norwegian gauging stations. We used the official EA rating curves to calculate stage for the high (H_HF_) and low (H_LF_) flow values as the stage corresponding to the means of the annual maxima and minima discharges obtained from the daily discharge time series. For mean flow, we used the stage corresponding to the mean of the discharge time series (over the period of record for each gauge). The 2.5th and 97.5th percentile discharge uncertainty bounds (i.e., the 2.5th and 97.5th percentiles from the Gaussian mixture model described above) were then used to define the upper ( ${\hat{Q}}_{U}^{95}$) and lower ( ${\hat{Q}}_{L}^{95}$) discharge uncertainty bounds for the low, mean and high discharge evaluation points. If there were multiple rating curves then an average of the stage values from the multiple curves were used. Note that Petersen‐Øverleir et al. instead used the mean daily stage time series for calculating the low, high and mean points on the rating curves, but we did not have stage time series data for all stations. Finally, relative uncertainties were quantified by calculating the width of the interval between the uncertainty bounds as a percentage of the discharge values from the official rating curve (Q_RC_). The equation for high flows is shown as an example:

(5) $$\frac{{\hat{Q}}_{U}^{95}\left(H_{HF}\right)-{\hat{Q}}_{L}^{95}\left(H_{HF}\right)\;}{Q_{RC}\left(H_{HF}\right)}\;x\;100$$

## 3 Results

### 3.1 Fitting Uncertainty Bounds for Different Stage‐Discharge Relationships

**Figure 5:**
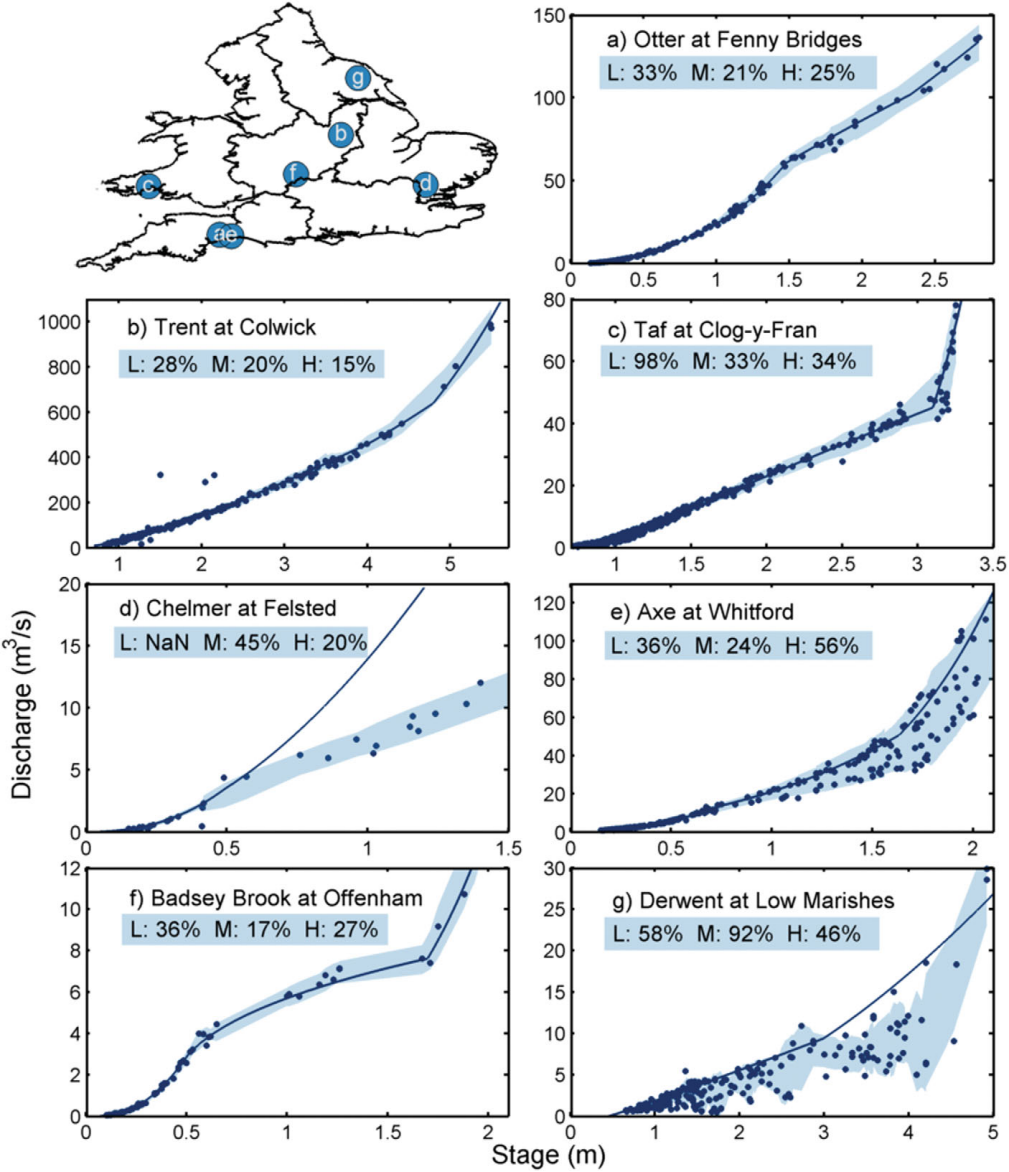
Examples from seven gauging stations of official EA rating curves, stage‐discharge measurements and their 95% uncertainty bounds calculated using the framework. Discharge uncertainties in percent for low (L), mean (M) and high (H) flow discharge are displayed in the shaded boxes.

**Figure 6:**
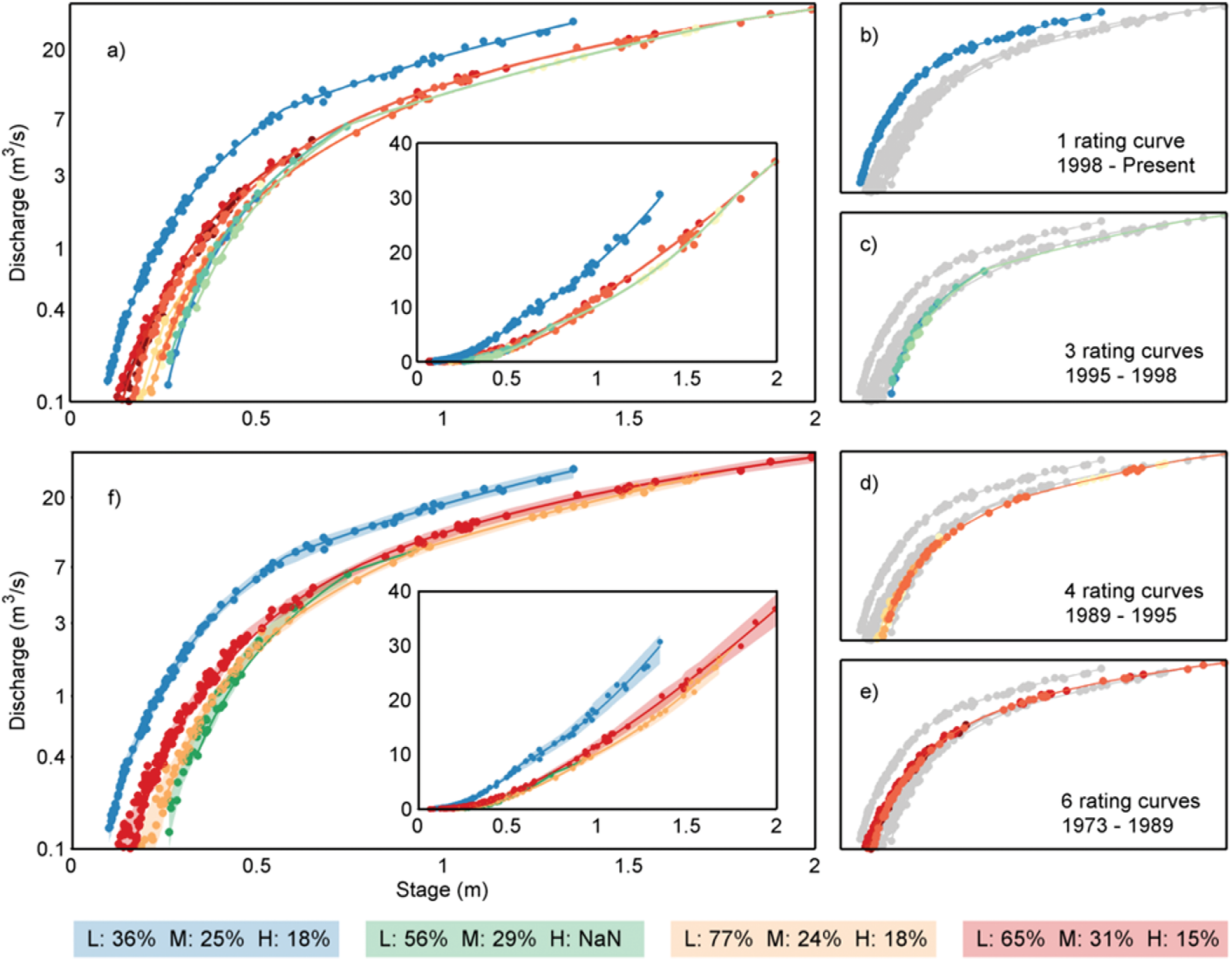
Stage‐discharge relationship for Taw at Taw Bridge from 1973 to 2014, (a) all rating curves and stage‐discharge measurements, main figure plotted using log(discharge) to emphasize low flows, inset figure plotted as untransformed discharge, (b–e) groups of rating curves with the dates where the rating curves were valid, and (f) final 95% uncertainty bounds for the four groups of rating curves, main figure plotted using log(discharge) to emphasize low flows, inset figure plotted as untransformed discharge. The discharge uncertainty values at low, mean and high flow for the four groups of rating curves are shown at the bottom of the plot.

To demonstrate the flexibility of the methodology, we present results from eight gauging stations (Figures 5 and 6) representing the different types of errors and stage‐discharge relationships identified in Figure 2. Figure 5 shows the official EA rating curves, stage‐discharge measurements, locations and estimated uncertainty bounds for seven different gauging stations. The figure demonstrates the applicability of the methodology for different types of gauging stations and stage‐discharge relationships, but also highlights one of the limitations of the framework. The results show that the discharge uncertainty magnitude varied between the gauging stations and over the flow range. There are examples of stable rating curves (Figure 5a), the ability of the methodology to robustly handle outliers (Figure 5b), changes in the stage‐discharge relationship due to out‐of‐bank flows (Figure 5c), deviations of stage‐discharge measurements to the rating curve (Figure 5d), gauging stations where the discharge uncertainty changes substantially at high flows (Figure 5e), multisection rating curves (Figure 5f) and an example where the span chosen automatically in the cross validation was not appropriate (Figure 5g). The resulting uncertainty bounds provide consistent results for most of the gauging stations where the uncertainty bounds reflect the scatter in the gauging data. For the majority of cases, the plots demonstrate that the window set in the cross‐validation procedure was not over or under‐fitting for any of the stage‐discharge relationships. Figures 5c and 5f show that the framework could handle complex stage‐discharge relationships with multiple sections and abrupt changes to the rating curve. Figure 5c (Taf at Clog‐y‐Fran) shows the stage‐discharge relationship for a velocity‐area station where the right flood bank overspills during peak flow events at 3.2–3.4m. As a result there is an abrupt change in the stage‐discharge relationship. This illustrates the need for high‐flow gaugings to assess uncertainties related to out‐of‐bank flows. Figure 5d (Chelmer at Felsted) illustrates a case where the assumption of a power‐law equation is not appropriate and shows that the framework can account for such non‐standard stage‐discharge relationships. In this case, the flows are heavily influenced by the downstream level and backwater is causing the flows to deviate significantly from the rating curve. For this station we were unable to calculate low flow uncertainty as there were no stage‐discharge measurements for low flows. The station in Figure 5e had considerable scatter at high flows and in contrast to the other six gauging stations the uncertainty was highest (in percentage terms) for high flows. This station was designed primarily for accuracy at low flows and although bankfull stage occurs at 2.1m, there is overspill at 1.65m on the left bank which may account for a large amount of the scatter. The resulting uncertainty bounds demonstrate that the methodology is able to capture this changing uncertainty by locally fitting the data and accounting for the scatter in the data. Finally, the station Low Marishes on the River Derwent (Figure 5g) highlights a case where the span automatically chosen in the cross‐validation procedure resulted in uncertainty bounds that are uneven and unrealistic as the LOWESS procedure has overfitted the stage‐discharge measurements. In such cases (for 23 out of the 500 stations), we selected an appropriate span manually rather than having one chosen by the cross‐validation procedure.

The framework also provided a measure of how the discharge uncertainty changes over time. Figure 6 shows one such example for the Taw Bridge gauging station on the river Taw. This particular station has 14 different rating curves (Figure 6a), 13 of which are associated with the old velocity‐area station that was built in 1973 and then 1 rating curve from 1998 when the gauging station was upgraded to a Flat V weir because of unstable bed control and multiple rating‐curve changes. Figures 6b–6e show the different groups of rating curves chosen by the maximum standardized deviation analysis (Section 2.3.2) and highlights its applicability for identifying replicates of the data. The final uncertainty bounds were then calculated for four subsets of the data (Figure 6f). As a result of the weir construction, the discharge uncertainty at low flows decreased significantly (see shaded boxes at the bottom of Figure 6). This demonstrates the value of separating the stage‐discharge measurements according to the individual rating curves, hence reflecting changes in the channel shape or other local conditions at the gauging station.

### 3.2 Benchmarking Discharge Data Quality in England and Wales

**Figure 7:**
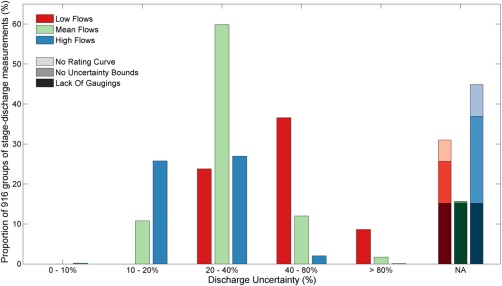
Relative discharge uncertainty results from the 916 groups of stage‐discharge measurements for low, mean and high flows. NA indicates the stations where discharge uncertainty for that particular flow value could not be calculated; the lightest shade for instances where the EA rating curve did not extend to that flow, the intermediate shade where the uncertainty bounds did not extend to that flow and then the darkest shade where there were too few stage‐discharge measurements to calculate discharge uncertainty for gauging stations with multiple groups of stage‐discharge measurements.

**Figure 8:**
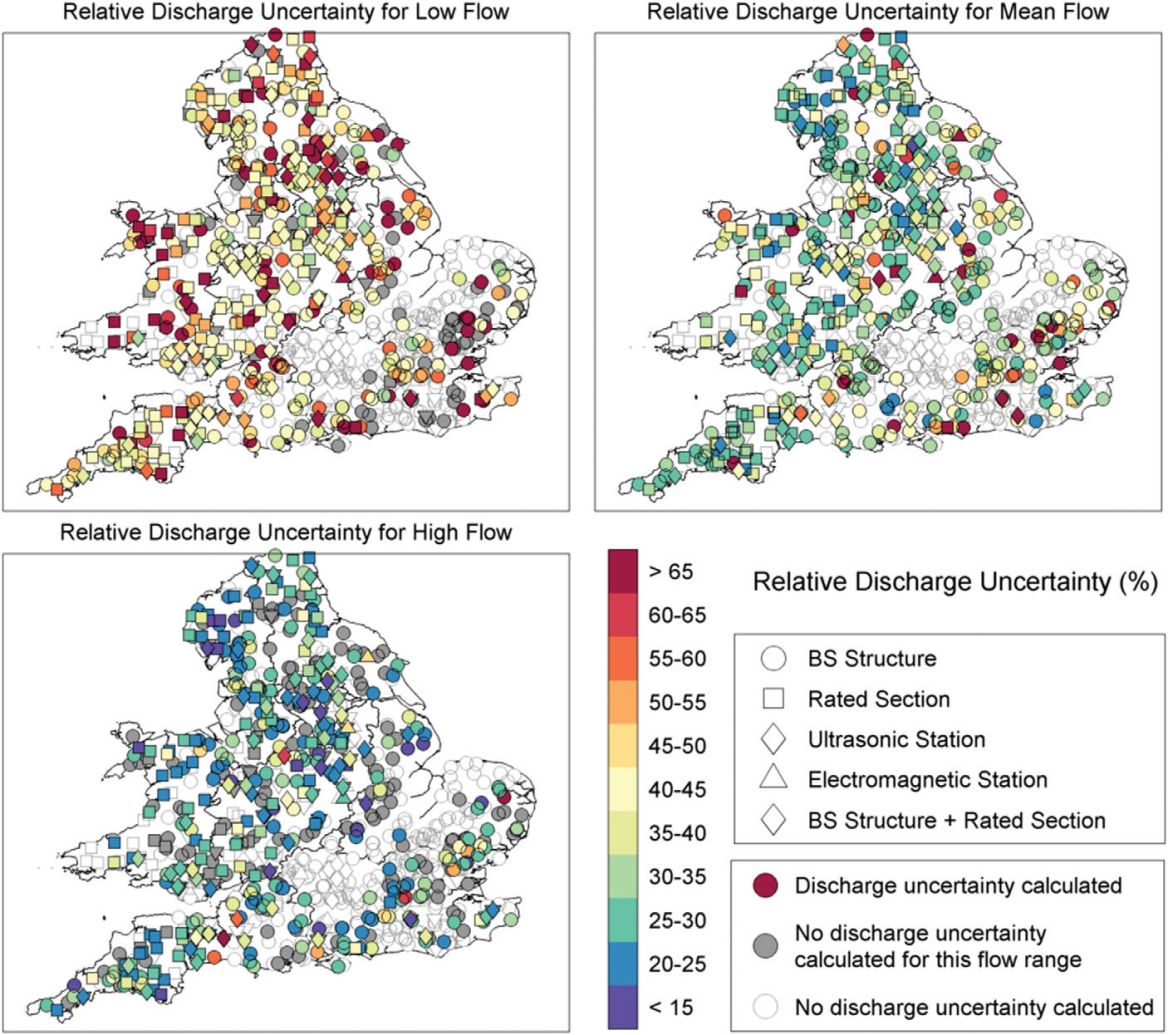
Maps showing the spatial pattern of discharge uncertainty for low, mean and high flows for the most recent group of stage‐discharge measurements for each station. An unfilled circle shows that discharge uncertainty was not calculated for that gauging station.

Figures 7 and 8 present an overview of the results from the discharge uncertainty analysis for the 500 gauging stations. Figure 7 displays results of the low, mean and high flow relative discharge uncertainty intervals (Equation (5)) for all the groups of stage‐discharge measurements (see Section 2.3.2, as there can be more than one group per station). The relative uncertainty intervals for the discharge evaluation points varied from 9 to 397% and there were distinct differences in the uncertainty for low, mean and high flows. Low‐flow uncertainties generally had the highest relative uncertainties varying from 20 to 397%. The highest relative discharge uncertainty interval (397%) occurred for a gauging station where the low flow value was 0.015m^3^/s meaning that the absolute discharge uncertainty is small (0.06m^3^/s) despite the high percentage. For low flows, 37% of the groups of stage‐discharge measurements fell within the 40–80% discharge uncertainty band, with 24% of the groups having a low‐flow uncertainty less than 40%. In contrast, 70% (53%) of the groups (83% (96%) discounting the groups where we were unable to calculate discharge uncertainty) had a mean (high) flow uncertainty interval of less than 40%. The high proportion of missing values for high flows (403 or 44% of the groups) occurred because the methodology was unable to extrapolate beyond the limits of the stage‐discharge measurements, and therefore reflects the lack of stage‐discharge measurements at high flows.

Figure 8 shows maps of the relative discharge uncertainties for the 500 gauging stations in England and Wales, with unfilled grey markers showing the rest of the gauging station network (910 stations) and thus where discharge uncertainty was not calculated. The discharge uncertainty intervals are shown for the most recent group of stage‐discharge measurements for each station. Results for historic ratings at stations with multiple rating curves are thus not shown. Interestingly, there were no clear spatial patterns in the discharge uncertainties, and we found no strong links between discharge uncertainty and gauging station type or region (results not shown). There was no clear relation between the spatial patterns for low, mean and high flows, showing that stations do not generally have consistently low or high discharge uncertainty across the flow range. The lack of gauging stations in the South‐East around the Thames is because most of the gauging stations in this region utilize weir calculations rather than rating curves and they had few stage‐discharge measurements which meant that our framework was not applicable.

## 4 Discussion

### 4.1 A Generalized Framework for Estimating Discharge Uncertainty

This study has developed a generalized framework for estimating discharge uncertainty and evaluated it nationally for gauging stations in England and Wales. Many studies have developed methods for estimating discharge uncertainty for different gauging stations and types of stage‐discharge relationships [*Guerrero et al*., 2012; *Le Coz et al*., 2014; *Reitan and Petersen‐Øverleir*, 2009; *Westerberg et al*., 2011a]. However, the pursuit of large‐sample hydrology [*Gupta et al*., 2014] together with a better understanding of the uncertainties and information content of data [*McMillan et al*., 2012] requires discharge uncertainty frameworks that can operate on minimal user and data input to quantify and benchmark discharge uncertainties over hundreds of gauging stations. We developed a novel framework that relies solely on the rating curve history and the gauging data, while requiring minimal user input to operate for hundreds of stations. Through nonparametric fitting of the data we were able to account for gauges that did not follow the traditional power‐law rating curve and handle multiple multisection rating curves robustly with little user input. The results show that the framework can represent place‐specific uncertainties by accounting for the different types of errors found in the stage‐discharge relationship at different gauging stations. A flexible framework is essential in regions such as England and Wales that exhibit a large diversity in the types of gauging stations, the controls and the errors sources that affect the stage‐discharge relationship.

### 4.2 Data Quality in England and Wales

Our results allow the quality of discharge data to be assessed nationally. We found that mean (high) flow relative discharge uncertainty was less than 40% for 83% (96%) of the gauging stations for the most recent group of stage‐discharge measurements. Similar to results from Norway [*Petersen‐Øverleir et al*., 2009], higher relative discharge uncertainties were found at low flows, which is not surprising given larger natural and measurement uncertainties for low‐flow conditions [*Petersen‐Øverleir et al*., 2009; *Tomkins*, 2014; *Westerberg et al*., 2011a]. However, our relative discharge uncertainty estimates for mean flow and high flow were generally higher, for example, there were no rating curves that had a mean flow uncertainty of less 10%, compared to nearly 10% of the rating curves in Norway [*Petersen‐Øverleir et al*., 2009]. Apart from differences in natural conditions, this could be due to the inclusion of measurement error in this study and the different calculation of the uncertainty bounds. In contrast, our high flow uncertainty estimates were lower; this is most likely because our framework did not allow for quantification of extrapolation uncertainties, which means that our analysis excluded many stations (section 3.2) with potentially large high‐flow uncertainty.

Despite regional differences in the type of gauging station, the number of historical rating curves and stage‐discharge measurements (Figure 1), discharge uncertainty was found to be highly place‐specific. This suggests that uncertainty at a given gauging station is mainly determined by a number of local site conditions including how the gauging station is managed, the frequency of flow gaugings, whether it has any backwater or weed growth problems, if the river overtops the banks, and if there is any bypassing of flow. These site conditions can result in large discharge uncertainty values, for example, 14% of the gauging stations had a relative discharge uncertainty at mean flow greater than 40%. The estimated uncertainties were generally not constant over time or over the flow range. This has important implications for hydrological modeling, flood modeling and water quality monitoring [*Lloyd et al*., 2015] within England and Wales; not only for studies focused on a single catchment but also for comparative catchment studies. Recognition of uncertainty in the stage‐discharge relationship is essential for robust assessments of hydrological models and to draw accurate conclusions in analyses based on discharge data. This is becoming increasingly recognized within hydrological research with recent studies incorporating discharge uncertainty into model diagnostics [*Coxon et al*., 2014; *Renard et al*., 2010; *Thyer et al*., 2009; *Westerberg and Birkel*, 2015], yet there is still progress to be made. Many studies that incorporate large numbers of catchments for hydrological model testing, development and evaluation [*Coron et al*., 2012; *Kollat et al*., 2012; *Perrin et al*., 2008; *Pushpalatha et al*., 2011; *Van Esse et al*., 2013] and regionalization [*Merz and Blöschl*, 2004; *Oudin et al*., 2008; *Parajka et al*., 2005; *Sefton and Howarth*, 1998; *Yadav et al*., 2007; *Young*, 2006] have yet to incorporate discharge uncertainty. Our study provides a first step toward that goal by providing a simple nonparametric framework to estimate discharge uncertainty locally at many gauging stations so it can be incorporated not only within hydrological modeling but any environmental modeling application that utilizes discharge data. While this is an important first step, there will need to be careful consideration of how these uncertainties are incorporated into modeling analyses and of how to provide discharge uncertainty estimates across the entire flow range when using this framework. Moreover, a complete uncertainty assessment will require large sample studies to include estimates of rainfall uncertainty and these important research questions will be the focus of our future studies.

### 4.3 Limitations and Future Developments

There are still some challenging cases where the resulting uncertainty bounds may not be appropriate and where the framework could be further developed. First, although the window size was chosen by a cross‐validation procedure, it did not always provide the best option for all sets of stage‐discharge measurements and the resultant uncertainty bounds could in some cases become uneven and unrealistic particularly when there was large scatter in the stage‐discharge data (Figure 5g). These cases were identified through visual inspection of the discharge uncertainty bounds and for the 23 stations where the uncertainty bounds were deemed unrealistic we selected the appropriate window size manually so that the LOWESS fit did not overfit or under‐fit the data.

Second, the LOWESS procedure is a data‐driven approach that depends on fitting the data locally within windows and it is therefore highly dependent on the available stage‐discharge data. This leads to two limitations in our approach. First, we set 20 stage‐discharge measurements as a minimum number to provide an estimate of discharge uncertainty (fewer than 20 measurements often led to poor LOWESS fits) and this meant that 152 gauging stations were discounted from this study. The framework is therefore currently not applicable in regions where the established practise is to make few gaugings. Second, the methodology is unable to extrapolate beyond the limits of the data and we were unable to calculate uncertainties at low (high) flows for 31% (44%) of the groups of stage‐discharge measurements. Care must be taken when extrapolating the stage‐discharge relationship as the estimated uncertainties may not be valid in some cases. This would in particular be the case where bankfull stage values are reached but no data exist to characterize the step change in the stage‐discharge relationship. Figure 2e provides a good example of how disinformative such extrapolations might become if the above bankfull stage‐discharge data were not available. Instead of extrapolating the expected stage‐discharge relationship for these gauging stations, hydraulic simulations can be used to gain insights about discharge uncertainties for extrapolated flows [e.g., *Di Baldassarre and Claps*, 2010].

Third, the estimate of the discharge measurement errors using the logistic distributions likely provides a better estimate than using (constant) literature values transferred from other studies, however, there are some limitations with this approach. Although the stage‐discharge measurements included in estimating the measurement uncertainties were taken over a long period (1962–2012) using different gauging techniques, we did not include information about measurement methods or how these have changed over time. The estimates may also not be equally representative across the flow range as we did not have any measurements for out‐of‐bank flows where a site‐specific step change in variance is expected, and there is potentially more error equifinality at low flows (although most of the stations we used had structures with good low‐flow control). There was only a small effect to the final LOWESS estimates at low flows of using a constant logistic measurement error equal to the high flow estimate across the whole flow range (results not shown). For the stage data a constant value of ±5 mm was assumed since we did not have further information about these errors. However, this estimate could be expected to vary significantly both spatially and temporally with larger uncertainties, for example, occurring during flow peaks if stage changes rapidly during the time of the gauging.

Finally, when using the framework to obtain the uncertainty bounds, the aim was to represent the uncertainty of the scatter in the stage‐discharge data since there are often important non‐random error sources affecting the data and information about these in the scatter. This means that the uncertainty bounds may be too wide if the scatter at a particular gauging station is instead dominated by random error sources in the observations of stage and discharge. However, the analyses of the 500 gauging stations in this study showed that non‐random error sources of varying origin were frequently present in the data (section 2.2). Without further site‐specific information about their magnitude or character it was therefore motivated to include the total data scatter in order to represent both aleatory and epistemic uncertainty sources and provide *robust outer estimates* for the discharge uncertainty. These results highlight the uncertainty about uncertainty intervals (or *uncertainty^2^*) in the presence of non‐negligible epistemic uncertainty sources, discussed and illustrated by *Juston et al*. [2014] in the context of rating‐curve uncertainty and hydrologic change detection. The mix of random and non‐random error sources could have significant consequences when transforming these uncertainty estimates back on to the discharge time series and this will be an important consideration when utilizing the framework in future studies.

## 5 Conclusions

This paper presents a versatile framework for assessing the discharge uncertainty at many gauging stations with a variety of different errors in the stage‐discharge relationship. The framework relies on information from the rating curves and stage‐discharge measurements and is based around three key components; (1) a robust nonparametric approach for fitting multiple rating curves, (2) a methodology for quantifying discharge measurement error, and (3) techniques to account for outliers in the data and scatter in the stage‐discharge data. It also uses information from the official EA rating curves to assess how discharge uncertainties change over time. To demonstrate its flexibility and applicability to many gauging stations we tested the framework on 500 gauging stations in England and Wales. Results show that the framework is robust for a number of different gauge types where the uncertainty bounds reflect the scatter in the stage‐discharge measurements well. An important outcome of this study is how local conditions at a gauging station determine the magnitude of discharge uncertainty and how the discharge uncertainty changes over the flow range and over time. These local conditions not only include natural changes such as weed growth but also the management of the gauging station such as how often the rating curve is updated. The analysis undertaken in this study could be extended by utilizing the framework to identify more/less reliable gauging stations and indicate priorities for hydrometry teams. Future research will aim to extend the framework to enable uncertainty estimates for extrapolated segments and assess discharge uncertainty at gauging stations with few stage‐discharge measurements.

## Supporting information

Supporting Information:

Supporting Information S1
