# Supplementary material for: A novel framework for discharge uncertainty quantification applied to 500 UK gauging stations
Source: Water Resour Res. 2015 Jul 19;51(7):5531–46. doi: 10.1002/2014WR016532 (PMC4755227; doi:10.1002/2014WR016532)

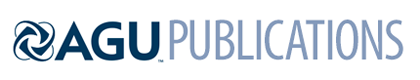


*Water Resources Research*

Supporting Information for

**A novel framework for discharge uncertainty quantification applied to 500 UK gauging stations**

G. Coxon^1^, J. Freer^1^, I. K. Westerberg^2,3^, T. Wagener^2^, R. Woods^2^, P. J. Smith^4^

(1) School of Geographical Sciences, University of Bristol, Bristol, UK, (2) Department of Civil Engineering, University of Bristol, Bristol, UK, (3) IVL Swedish Environmental Research Institute, Stockholm, Sweden, (4) Lancaster Environment Centre, Lancaster University, Lancaster, UK

**Contents of this file**

Text S1

Figures S1 to S3

**Additional Supporting Information (Files uploaded separately)**

None

**Introduction**

The supporting information provides additional insights on the sensitivity of the results to methodological choices presented in the main text. In particular, we address the sensitivity of the results to the number and distribution of stage-discharge measurements.

Text S1. Sensitivity of the results to the number and distribution of stage-discharge measurements

To assess the sensitivity of the framework to the number and distribution of stage-discharge measurements, we conducted two tests with three gauging stations. We assessed the fit of the LOWESS rating curves and the discharge uncertainty estimates to variations in the number and distribution of the stage-discharge measurements.

To achieve this, we conducted these tests on three gauging stations which were chosen to represent different types of stage discharge relationships:

1) *Manifold at Ilam*, little scatter in stage-discharge measurements and good coverage of the rating curve,

2) *Axe at Whitford,* large scatter in stage-discharge measurements and good coverage of the rating curve,

3) *Badsey Brook at Offenham*, multi-segment rating curve and uneven distribution of the stage-discharge measurements over the rating curve.

For the first test, the number of stage-discharge measurements was varied by randomly re-sampling from the full number of stage-discharge measurements for the three gauging stations. The number of stage-discharge measurements was increased from five to the total number of stage-discharge measurements by random sampling without replacement. The first five stage-discharge measurements always included the lowest and highest stage gauging to allow comparison between the sampled and true result. This process was repeated 100 times to provide 100 different random samples of stage-discharge measurements. For the second test, the stage-discharge measurements were randomly sampled from five bins of stage-discharge measurements where each bin contained an equal number of stage-discharge measurements. The measurements were sorted by stage into the five bins and 100 different random samples were created as before.

To evaluate the results, we compared the LOWESS fits and the discharge uncertainty estimates from each sub-sampled set of stage-discharge measurements against the LOWESS fits and discharge uncertainty estimates from the full set of stage-discharge measurements. Each of the LOWESS fits was normalized by the highest value of discharge from the stage-discharge measurements and then compared to the ‘true’ normalized LOWESS fit created by using all the stage-discharge measurements. The normalized differences were averaged across the flow range to provide a single estimate of the difference between the LOWESS fits for each sub-sampled dataset. We also calculated the width of the uncertainty bounds at every stage point and compared these to the ‘true’ estimates of discharge uncertainty derived from all the stage-discharge measurements. The mean of the differences between the sub-sampled and true estimate is shown in Figures S1-3.

The results from this analysis show that the framework is sensitive to the number and distribution of stage-discharge measurements. Although the framework could be applied to any gauging station with at least five stage-discharge measurements, large differences occur where there are less than 10 stage-discharge measurements and the points are randomly sampled across the flow range. Smaller differences are found when the points are sampled from the five bins. There are also differences between the catchments. Larger differences in both the fit of the rating curve and the discharge uncertainty estimates are found when there is large scatter in the stage-discharge measurements or there is a multi-section rating curve. For complex multi-section rating curves, more data would be required to ensure a good fit.

In the main paper we utilized 20 stage-discharge measurements as the minimum number of stage-discharge measurements needed to estimate discharge uncertainty. This was a good choice for each of the three example stations and resulted in average differences of around 10% or smaller.

Figure S1. LOWESS fits with different numbers of stage-discharge measurements for a) random sample d) stage-discharge measurements sampled from five bins for the Manifold at Ilam gauging station. Mean normalized difference between sampled LOWESS fit and ‘true’ estimate for b) random sample e) stage-discharge measurements sampled from five bins. Each line represents one of the 100 different random samples. Mean percentage difference between sampled discharge uncertainty estimates and ‘true’ discharge uncertainty estimates for c) random sample f) stage-discharge measurements sampled from five bins. The y-axis range is capped at 50%. Each black line represents one of the 100 different random samples, the red line represents the mean of the random samples and the grey dashed line marks twenty stage-discharge measurements that was selected in the paper.


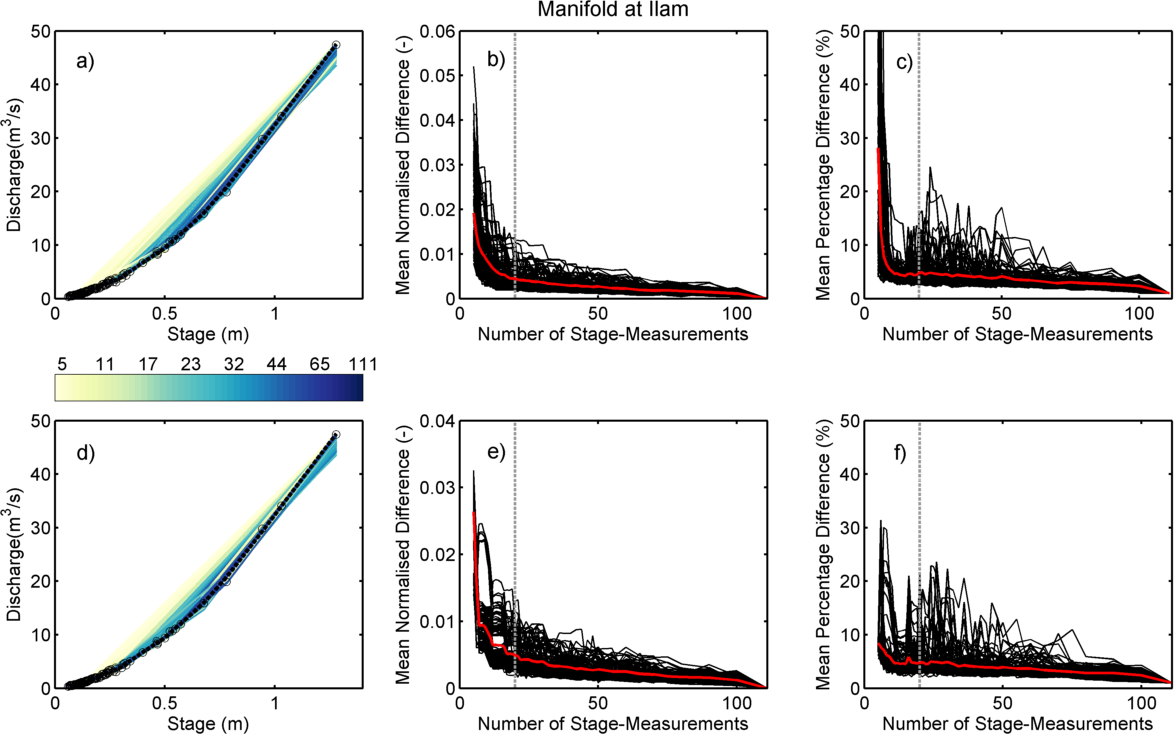


Figure S2. LOWESS fits with different numbers of stage-discharge measurements for a) random sample d) stage-discharge measurements sampled from five bins for the Axe at Whitford gauging station. Mean normalized difference between sampled LOWESS fit and ‘true’ estimate for b) random sample e) stage-discharge measurements sampled from five bins. Each line represents one of the 100 different random samples. Mean percentage difference between sampled discharge uncertainty estimates and ‘true’ discharge uncertainty estimates for c) random sample f) stage-discharge measurements sampled from five bins. The y-axis range is capped at 50%. Each black line represents one of the 100 different random samples, the red line represents the mean of the random samples and the grey dashed line marks twenty stage-discharge measurements that was selected in the paper.


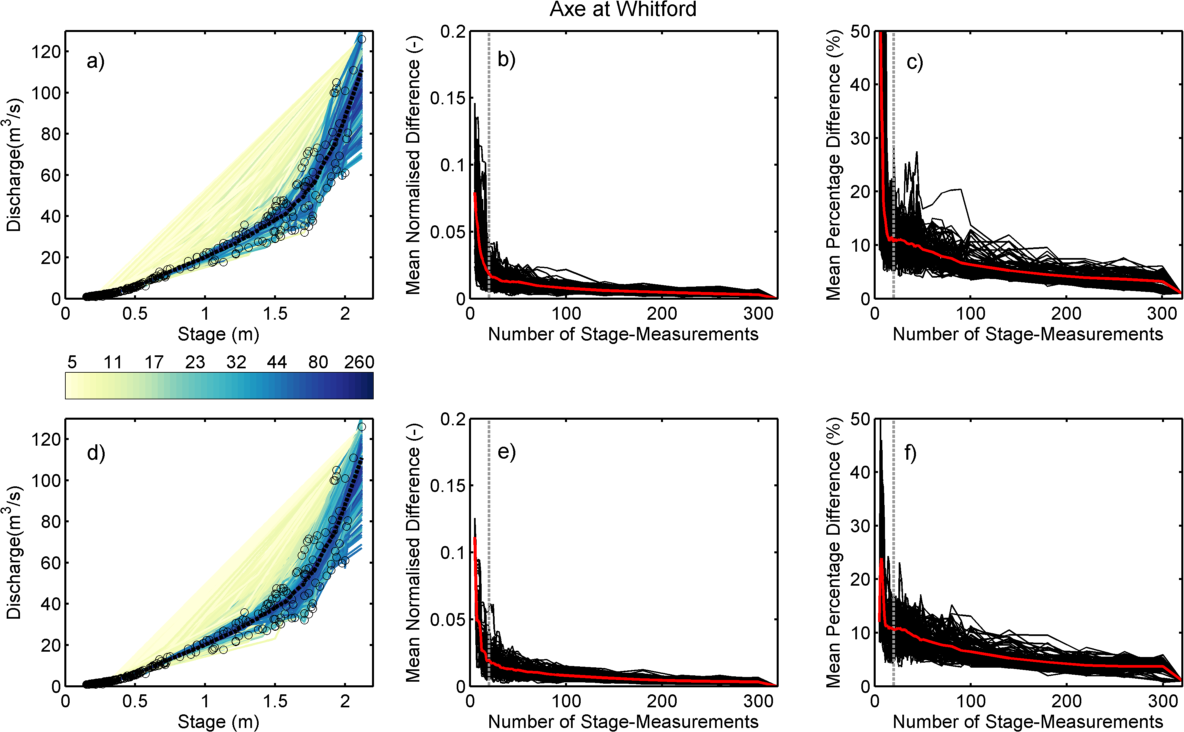


Figure S3. LOWESS fits with different numbers of stage-discharge measurements for a) random sample d) stage-discharge measurements sampled from five bins for the Badsey Brook at Offenham gauging station. Mean normalized difference between sampled LOWESS fit and ‘true’ estimate for b) random sample e) stage-discharge measurements sampled from five bins. Each line represents one of the 100 different random samples. Mean percentage difference between sampled discharge uncertainty estimates and ‘true’ discharge uncertainty estimates for c) random sample f) stage-discharge measurements sampled from five bins. The y-axis range is capped at 50%. Each black line represents one of the 100 different random samples, the red line represents the mean of the random samples and the grey dashed line marks twenty stage-discharge measurements that was selected in the paper.


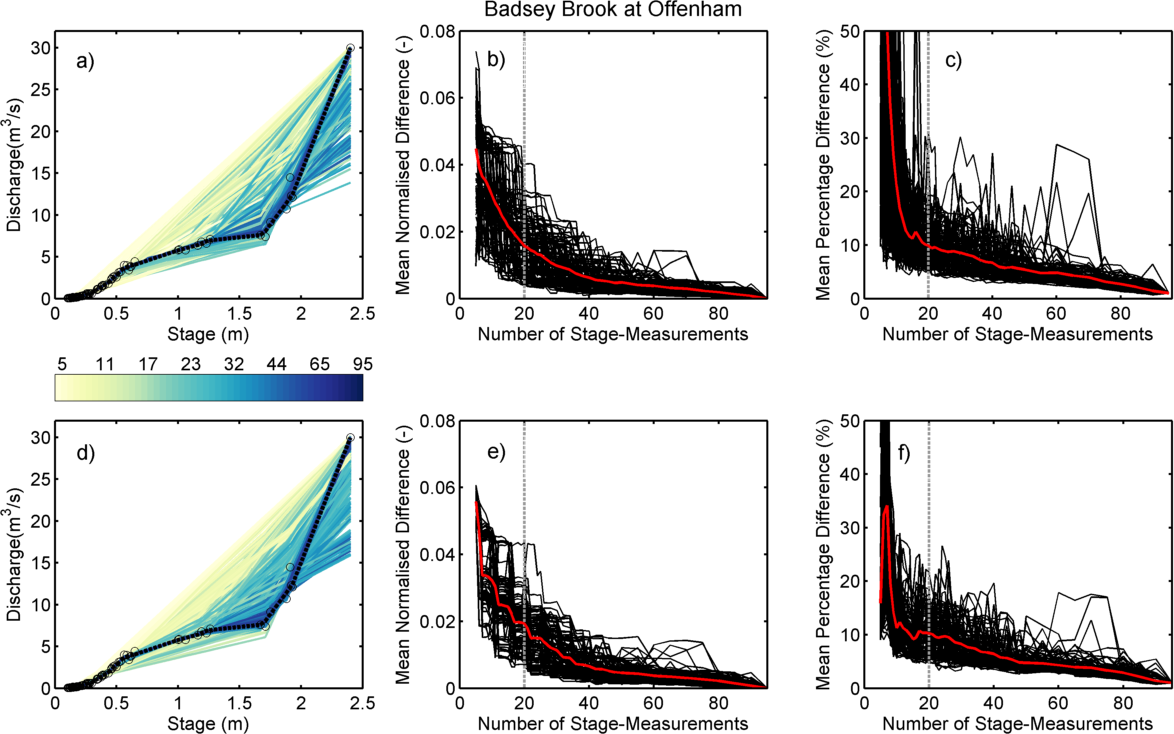

Supplement: Supplementary file 1 — Supporting Information S1 [file WRCR-51-5531-s001.doc]
